# Inhibition of the checkpoint kinase Chk1 induces DNA damage and cell death in human Leukemia and Lymphoma cells

**DOI:** 10.1186/1476-4598-13-147

**Published:** 2014-06-10

**Authors:** Christopher Bryant, Kirsten Scriven, Andrew J Massey

**Affiliations:** 1 Vernalis R&D Ltd, Granta Park, Cambridge CB21 6GB, UK

**Keywords:** Chk1, Leukemia, Lymphoma, Kinase inhibitor, V158411

## Abstract

**Background:**

Chk1 forms a core component of the DNA damage response and small molecule inhibitors are currently being investigated in the clinic as cytotoxic chemotherapy potentiators. Recent evidence suggests that Chk1 inhibitors may demonstrate significant single agent activity in tumors with specific DNA repair defects, a constitutively activated DNA damage response or oncogene induced replicative stress.

**Methods:**

Growth inhibition induced by the small molecule Chk1 inhibitor V158411 was assessed in a panel of human leukemia and lymphoma cell lines and compared to cancer cell lines derived from solid tumors. The effects on cell cycle and DNA damage response markers were further evaluated.

**Results:**

Leukemia and lymphoma cell lines were identified as particularly sensitive to the Chk1 inhibitor V158411 (mean GI_50_ 0.17 μM) compared to colon (2.8 μM) or lung (6.9 μM) cancer cell lines. Chk1 inhibition by V158411 in the leukemia and lymphoma cell lines induced DNA fragmentation and cell death that was both caspase dependent and independent, and prevented cells undergoing mitosis. An analysis of *in vitro* pharmacodynamic markers identified a dose dependent decrease in Chk1 and cyclin B1 protein levels and Cdc2 Thr15 phosphorylation along with a concomitant increase in H2AX phosphorylation at Ser139 following V158411 treatment.

**Conclusions:**

These data support the further evaluation of Chk1 inhibitors in hematopoietic cancers as single agents as well as in combination with standard of care cytotoxic drugs.

## Background

The serine-threonine checkpoint kinases Chk1 and Chk2 form central key components in the DNA damage signaling response (DDR) [1]. Activation of the DDR results in a number of cellular responses including checkpoint activation and cell cycle arrest, initiation of DNA repair, regulation of transcription and apoptosis. The DDR can be activated by a range of endogenous and external insults including therapies currently used for the treatment of cancer such as ionizing radiation and cytotoxic chemotherapeutic agents such as gemcitabine, irinotecan and cisplatin [2,3]. Despite their similarity in name, Chk1 and Chk2 differ substantially in the structure of their kinase pocket [4,5] and in their cellular function with Chk1 suggested to be the major component responsible for responses to DNA damage [3,6,7]. Inhibiting Chk1 following genotoxic stress (such as that induced by cytotoxic chemotherapy) results in checkpoint abrogation, inhibition of DNA repair and induction of cell death in cells with a defective p53 response [8,9]. Small molecule inhibitors of predominantly the Chk1 kinase have been readily sought as a mechanism through which the anti-tumor activity of cytotoxic chemotherapeutics may be increased whilst sparing the normal cells [10-12]. This approach is currently being tested in the clinic with a variety of agents including LY2603618 [13], MK-8776 [14], GDC-0425 and GDC-0575 in combination with a range of standard of care chemotherapy drugs.

Evidence has begun to emerge that small molecule Chk1 inhibitors may have significant single agent activity in cancer cells with specific underlying genetic defects. This is often defined as a synthetic lethal relationship [15,16]. These can so far be defined as having specific defects in DNA-damage repair or response components, or are constitutively dependent on the DDR to complete an unperturbed round of DNA replication. The Fanconi Anemia (FA) pathway is a DNA repair pathway that is responsible for repairing crosslinked DNA [17]. Components of the FA pathway has been found to be lost or defective in a range of human cancers and are characterized by hypersensitivity to DNA crosslinking agents, chromosomal instability and reliance on DNA repair mediated by ATM. FA deficient cell lines were found to be sensitive to Chk1 silencing by siRNA compared to FA proficient cells [18]. Patients with complex karyotype acute myeloid leukemia (AML) had high levels of constitutive DNA damage (including high levels of pH2AX) and checkpoint activation. AML blast cells derived from these patients were sensitive to Chk1 siRNA or the kinase inhibitor UCN-01 compared to normal granulomonocyte progenitors [19]. Sensitivity to Chk1 inhibition has also been linked to replicative stress in a number of cancer cell types. In neuroblastoma cell lines, an siRNA screen identified siRNAs against Chk1 as the most potent inducers of cytotoxicity [20]. Chk1 mRNA expression was higher in MYC-Neuroblastoma-related (MYCN) amplified cancers and Chk1 was found to be phosphorylated on the auto-phosphorylation site Ser296 and the ATM activation site Ser345 in the absence of exogenous DNA damage insults. Neuroblastoma cell lines were found to be more sensitive to two Chk1 inhibitors SB21807 and TCS2312 compared to three non-neuroblastoma cancer cell lines. Sensitivity to SB21807 correlated with MYCN protein levels. Inhibition of Chk1 with the small molecule inhibitor AR678 inhibited the proliferation of a range of melanoma cell lines with low nM efficiency *in vitro*. The cytotoxicity of AR678 was suggested to be due to inhibition of S-phase Chk1 and failure of cytotokinesis or induction of apoptotic death and sensitivity correlated with levels of endogenous DNA damage most likely induced by replicative stress [21].

We utilized our own novel, potent, selective small molecule inhibitor of Chk1, V158411, to screen cell lines from a range of cancer types in an effort to identify additional tumor types for which single agent Chk1 inhibitor therapy may prove a rational treatment option.

## Results

### Pharmacological inhibition of Chk1 is cytotoxic in leukemia and lymphoma cell lines

Emerging evidence suggests that inhibiting the checkpoint kinase Chk1, in addition to potentiating cytotoxic chemotherapeutic agents, may exhibit single agent activity in cancers with underlying DNA repair, DNA damage response or DNA replication defects. We used the highly selective, potent checkpoint kinase inhibitor V158411 as a tool to identify cancer types where checkpoint inhibition may be a rationale therapeutic option.

**Figure 1:**
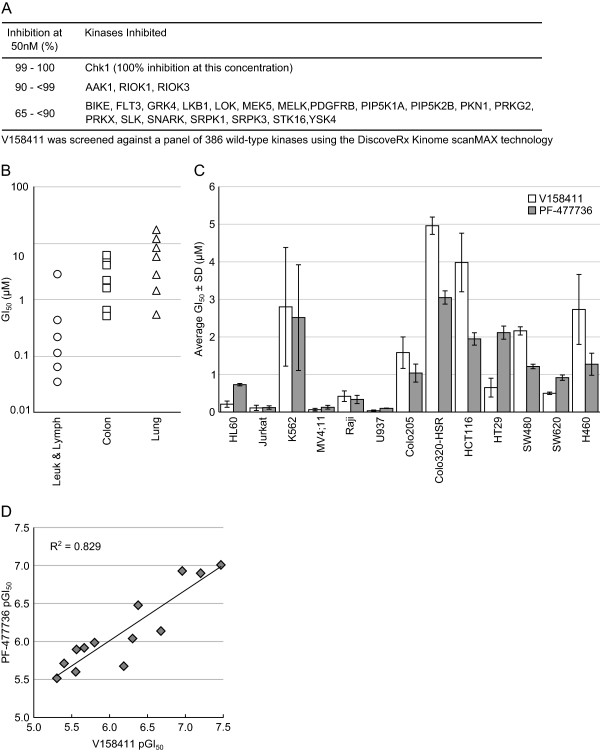
**Inhibition of Chk1 inhibits cell proliferation in human leukemia and lymphoma cell lines. (A)** Kinase selectivity profile of V158411. V158411 was screened at 50 nM, duplicate single-point against a panel of 386 wild-type kinases using DiscoveRx KINOME scanMAX technology. **(B)** V158411 inhibited the proliferation of leukemia and lymphoma cell lines compared to colon and lung cancer cell lines following 72 hour exposure to the drug. Values are the average of at least 4 determinations. **(C)** Comparison of V158411 and PF-477736 anti-proliferative activity against leukaemia and lymphoma cell lines compared to colon and lung cancer cell lines following 72 hour exposure to the drug. Values are the average of at least 4 determinations ± SD. **(D)** Regression analysis of V158411 and PF-477736 GI_50_ values. GI_50_ values from Figure 1**C** were converted to pGI_50_ values and plotted. R^2^ value was calculated using a linear trendline in Excel.

V158411 is a novel, potent, selective inhibitor of recombinant Chk1 and Chk2 kinases *in vitro* with IC_50_s of 3.5 and 2.5 nM respectively [22]. Against a panel of 386 kinases in a wide panel binding assay, V158411 inhibited the activity of one kinase (Chk1) in the range 99 – 100%, three kinases 90 – 99% and 19 kinases 65 – 90% at 50 nM (Figure 1A). In p53 defective HT29 cells, V158411 inhibited the etoposide induced auto-phosphorylation of Chk1 on Ser296 with an IC_50_ of 48 nM and Chk2 on Ser516 with an IC_50_ of 904 nM indicating a 19-fold cellular selectivity for Chk1 over Chk2. V158411 potentiated cytotoxic chemotherapy in p53 defective cancer cells *in vitro* and *in vivo*.

**Figure 2:**
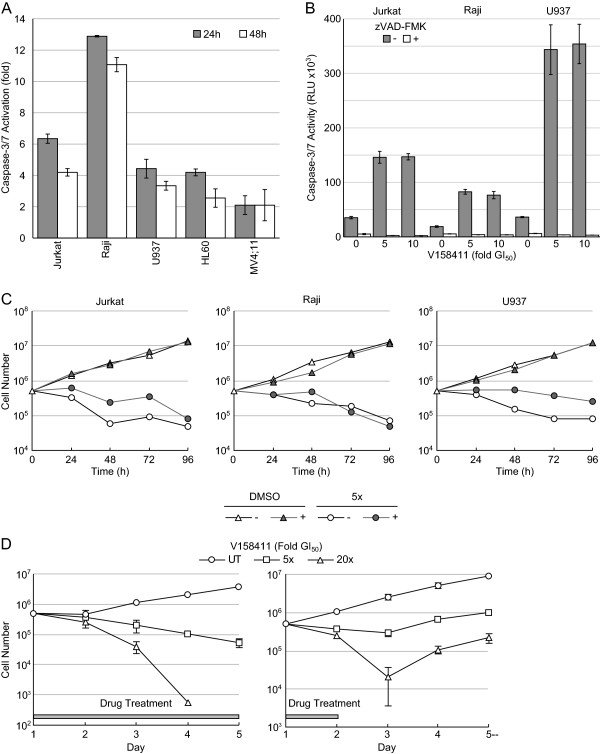
**V158411 induces caspase activation and cell death in human leukemia and lymphoma cell lines. (A)** Treatment with 5-times the GI_50_ of V158411 for 24 or 48 hours activated caspase-3/7 dependent apoptosis. Values are the average of 3 determinations ± SD. **(B)** zVAD-FMK blocks V158411 induced caspase 3/7 activation. Cells were treated with 5- or 10-times the GI_50_ of V158411 in the absence or presence of 25 μM zVAD-FMK. Values are the average of 3 determinations ± SD. **(C)** Cell death in leukemia and lymphoma cell lines induced by V158411 can occur independently of caspase-3/7 activity. Cells were treated with 5-times the GI_50_ of V158411 in the absence or presence of 25 μM zVAD-FMK for 24 hours. Values are the average of 3 determinations; error bars have been removed for clarity. **(D)** Continual treatment is more effective in inducing cell death in U937 lymphoma cells than pulsed treatment. U937 cells were treated with 5- or 20-times the GI_50_ of V158411 either continually for 96 hours (left) or a 24 hour pulse (right). Values are the average of 3 determinations ± SD.

In a screen of cell lines, V158411 inhibited the proliferation of five out of six of the leukemia and lymphoma cell lines tested with an average GI_50_ of 0.17 μM (Figure 1B and 1C, and Table 1) following 72 hour exposure to the drug. In comparison, the average GI_50_ for the seven colon cancer cell lines and for the seven lung cancer cell lines were 2.8 μM and 6.9 μM respectively. To confirm this, a second Chk1 inhibitor PF-477736 [23] was also profiled. As was observed for V158411, PF-477736 selectively inhibited the proliferation of the same five leukemia and lymphoma cell lines with an average GI_50_ of 0.28 μM (Table 1) compared to 1.7 μM for one lung and six colon cancer cell lines (Figure 1C). There was a close correlation between the sensitivity of a given cell line to V158411 and PF-477736 (R^2^ = 0.829, Figure 1D). Inhibition of cell proliferation was accompanied by a rapid and sustained increase in caspase-3/7 dependent apoptosis in all five hematopoietic cancer cell lines (Figure 2A). This was especially marked in the Raji and Jurkat cell lines where treatment with 5-times the GI_50_ of V158411 increased caspase-3/7 levels 13- and 6-fold respectively after 24 hours. Cell death induced by Chk1 inhibition could, however, occur independently of caspase-3/7 activity. The caspase-3/7 inhibitor zVAD-FMK effectively blocked V158411 induced caspase-3/7 activation (Figure 2B). Jurkat, Raji or U937 cells treated with V158411 still underwent cell death in the presence of the general caspase inhibitor zVAD-FMK (Figure 2C). In both Jurkat and U937 but not Raji cells, more cell death was observed in the absence of zVAD-FMK but was not blocked completely by zVAD-FMK. This therefore suggests that Chk1 inhibition in leukemia and lymphoma cells can induce cell death through a variety of cell death pathways. To further understand the effects of Chk1 inhibition on cell proliferation, two different drug exposure regimes were compared in U937 cells. A single 24 hour pulse of 0.7 μM V158411 reduced the fraction of viable U937 cells by 96% 24 hours after the end of treatment (Figure 2D). However, the viable population rebounded rapidly with the number of viable U937 cells increased 5.5-fold and 11-fold compared to the 24 hour cell count 48 and 72 hours after the end of treatment. Continual exposure to 0.7 μM V158411 for 72 hours had a more marked and permanent effect on U937 survival reducing the fraction of viable U937 cells by 99.9%.

**Table 1:** Growth inhibition of leukemia and lymphoma cell lines by V158411 and PF-477736

**Cell line**	**p53 status**	**GI**_**50 **_**(μM) ± SD**		**Tumor type**
		**V158411**	**PF-477736**	
HL60	Mut	0.21 ± 0.083	0.73 ± 0.033	Promyelocytic Leukemia
Jurkat	Mut	0.12 ± 0.07	0.12 ± 0.046	T-cell Lymphoma
K562	Mut	2.8 ± 1.6	1.9 ± 1.4	Chronic Myelogenous Leukemia
MV4-11	WT	0.063 ± 0.033	0.13 ± 0.051	Acute Monocytic Leukemia
Raji	Mut	0.42 ± 0.14	0.33 ± 0.11	Burkett’s Lymphoma
U937	Mut	0.034 ± 0.018	0.099 ± 0.006	Histiocytic Lymphoma

Mut, mutant; WT, wild type. GI_50_ values are the mean of n ≥ 4 ± SD.

### Inhibition of Chk1 induces DNA fragmentation and prevents entry into mitosis

**Figure 3:**
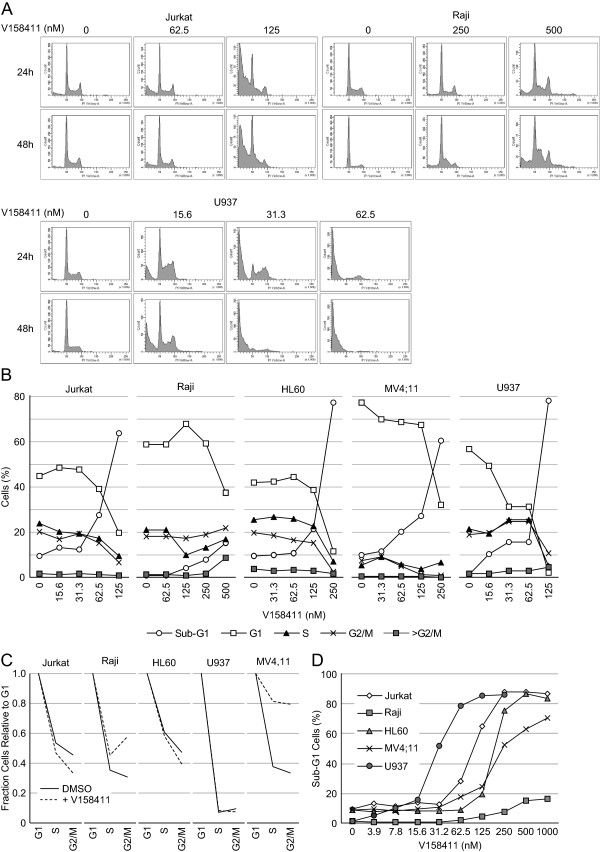
**Cell cycle changes induced in leukemia and lymphoma cells by V158411. (A)** Cell cycle profiles of Jurkat, Raji and U937 cells were determined by PI staining following treatment with the indicated concentrations of V158411 for 24 or 48 hours. **(B)** Quantification of the cell cycle changes observed after 24 hour treatment to V158411. **(C)** Comparison of the fraction of cells in G2 and M relative to G1 in cells treated with DMSO or the concentration of V158411 approximately equivalent to the GI_50_ for 24 hours. **(D)** V158411 treatment for 24 hours increased the percentage of leukemia and lymphoma cells with a sub-G1 DNA content.

**Figure 4:**
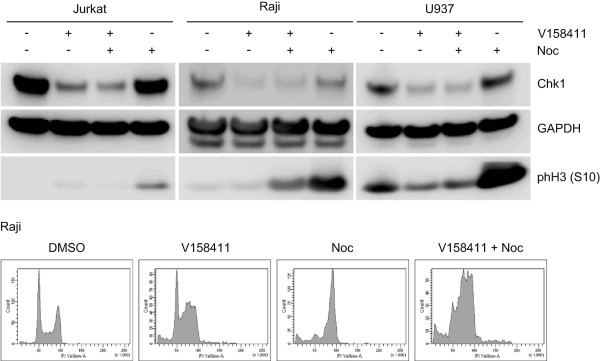
**Inhibition of Chk1 by V158411 prevents entry into mitosis.** Jurkat, Raji or U937 cells were treated with 1 μM V158411 in the presence or absence of 0.1 μM nocodazole for 24 hours. Changes to protein markers were evaluated by western blotting.

Pharmacological inhibition of Chk1 with V158411 did not induce a definitive cell cycle arrest in the five sensitive hematopoietic cancer cell lines (Figure 3A and B). However, changes in the ratio of cells in G1:S:G2/M was observed in 3 of the 5 cell lines. In Jurkat cells, V158411 treatment reduced the fraction of cells in S and G2/M relative to G1. In Raji and MV4;11, the fraction of cells in S and G2/M was increased by V158411 treatment. This was most noticeable for the MV4;11 cells (Figure 3C). V158411 induced a dose dependent increase in the fraction of cells with a sub-G1 DNA content. In Jurkat, HL60 and U937 cells, this accounted for nearly 90% of the cell population at the higher concentrations (Figure 3D). This is highly indicative of DNA fragmentation and cell death via apoptosis. To evaluate if cells were progressing into mitosis and undergoing death via mitotic catastrophe, we utilized nocodazole to trap cells in mitosis. Treatment of Jurkat, Raji or U937 cells with nocodazole led to an increase in the fraction of cells in mitosis as evidenced by an increase in the levels of phH3 (S10). Treatment with V158411 prevented cells progressing through the cell cycle and becoming arrested in mitosis by nocodazole (Figure 4). The addition of nocodazole did not prevent the V158411 induced degradation of Chk1.

### Chk1 inhibition induces Chk1 degradation and H2AX phosphorylation

**Figure 5:**
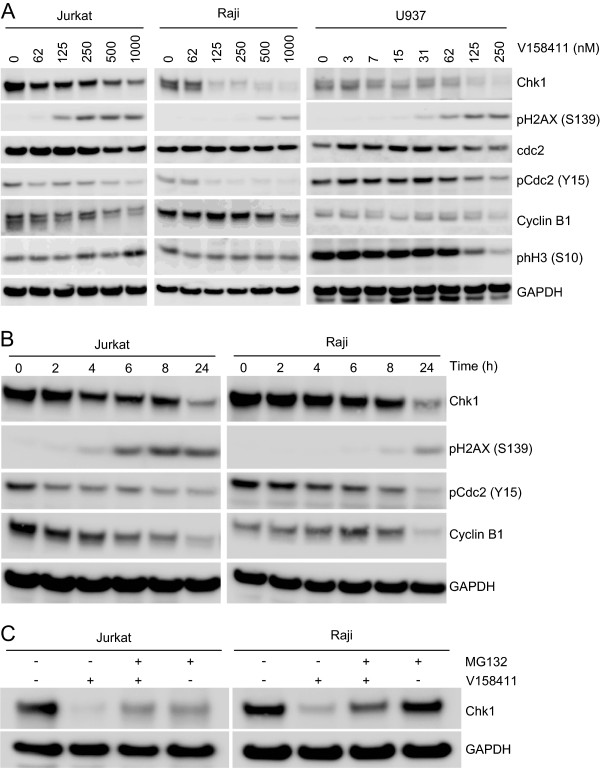
**Chk1 inhibition by V158411 induces Chk1 degradation and H2AX phosphorylation. (A)** Cells were treated with the indicated concentrations of V158411 for 24 hours. **(B)** Jurkat and Raji cells were treated with 1 μM V158411 for the indicated times. **(C)** Jurkat and Raji cells were treated with 1 μM V158411 in the presence or absence of 10 μM MG132 for 24 hours. Protein expression was characterized by western blotting with GAPDH used as a loading control.

The effects of V158411 on biomarker changes in Jurkat, Raji and U937 cells was evaluated. Treatment of all three cell lines with V158411 for 24 hours lead to a dose dependent decrease in Chk1 protein levels and a concomitant increase in the amount of H2AX phosphorylated at Ser139 (Figure 5A). In addition, the levels of Cdc2 phosphorylated at Tyr15 and total cyclin B1 were also reduced albeit at higher doses of V158411 than those needed to reduce Chk1 and induced pH2AX. In U937 cells, a reduction in the amount of Histone H3 phosphorylation on Ser10 could be observed. A time course of V158411 treatment in Jurkat and Raji cells indicated that maximal Chk1, cyclin B1 and pCdc2 (Y15) reduction occurred after 24 hours (Figure 5B). The kinetics of pH2AX induction differed between the two cell lines with an increase in pH2AX observed after 6 hours in Jurkat but not until 24 hours in Raji cells. The duration of pH2AX induction in Jurkat cells was maintained for at least 24 hours. In both cells lines, the degradation of Chk1 was dependent on the presence of a functioning proteasome. The proteasome inhibitor MG132 inhibited the V158411 induced degradation of Chk1 in Jurkat and Raji cells (Figure 5C).

### Western blot analysis of leukemia and lymphoma cell lines

**Figure 6:**
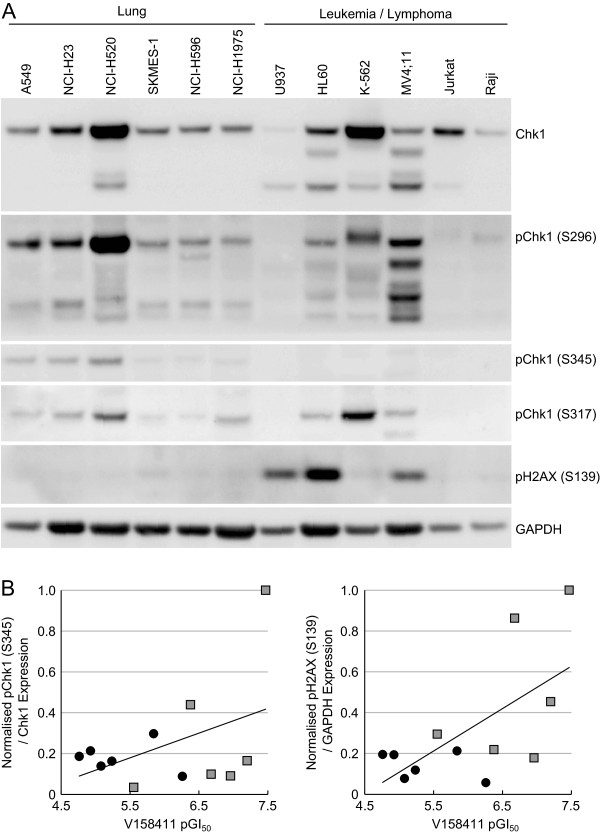
**Leukemia and lymphoma cell lines do not exhibit high endogenous expression levels of phosphorylated Chk1. (A)** Untreated whole cell protein extracts were prepared from the indicated cell lines and the expression levels of various protein markers determined by western blotting. **(B)** Protein expression was quantified by densitometry and correlated with the cell line sensitivity to V158411. Circles, lung cell lines; squares, leukemia and lymphoma cell lines.

In order to further understand the underlying mechanism for sensitivity of Leukemia and Lymphoma cells to the Chk1 inhibitors and identify biomarkers that may be potentially useful for identifying sensitive patients in clinical studies, we examined the expression levels and phosphorylation status of Chk1 in these cell lines by western blotting and compared it to a panel of six lung lines (Figure 6). Chk1 expression levels varied across the cell lines with the highest expression levels identified in NCI-H520 and K562 cells and very low levels in U937 and Raji cells. Phosphorylation of Chk1 on either serine 296, 317 or 345 was highly variable across the cell lines investigated. No correlation between Chk1 expression levels or phosphorylation on serine 296, 317 or 345 and sensitivity to the Chk1 inhibitors could be identified. There was an apparent increased basal expression level of pChk1 (S345) in lung cancer cell lines especially A549, NCI-H23 and NCI-H520 but this was not significantly higher than the leukemia and lymphoma cell lines (*P* = 0.02). There was no correlation between pChk1 (S345) expression levels and sensitivity to V158411 (Figure 6B, R^2^ = 0.186). In three of the Chk1 inhibitor sensitive leukemia/lymphoma cell lines, U937, HL-60 and MV4-11, the endogenous levels of H2AX phosphorylated on Ser139 was much higher compared to all other cell lines. Across the whole panel of cell lines analyzed, there was a weak correlation (R^2^ = 0.404) between pH2AX (S139) expression and V158411 sensitivity but no correlation (R^2^ = 0.250) when just the leukemia and lymphoma subset of cell lines were analyzed (Figure 6B).

## Discussion

Small molecule inhibitors of the checkpoint kinase Chk1 are currently undergoing early stage clinical evaluation in combination with DNA damaging cytotoxic chemotherapeutic drugs such as irinotecan and gemcitabine. Recent studies have started to identify cancer types sensitive to Chk1 inhibition as single agents; that is, in the absence of a cytotoxic chemotherapeutic drug. RNAi studies have identified neuroblastoma [20] and Fanconi’s Anemia [18] whilst small molecule inhibitor studies have revealed triple-negative breast cancer [24] and an Eμ-myc driven model of lymphoma as potential clinical targets of Chk1 inhibitor therapy [25,26]. Here we further extend this list of cancer types sensitive to Chk1 inhibitors as single agents to include cancers of a hematopoietic origin.

Treatment of a diverse range of leukemia and lymphoma cell lines with the selective Chk1 inhibitors V158411 or PF-477736 potently inhibited the proliferation of these cell lines and induced cell death that was both caspase-3/7 dependent and independent. This coincided with a reduction in the fraction of cells with a G1 DNA content and an increase in sub-G1 DNA content along with reduction in Chk1 protein levels and increased phosphorylation of H2AX on serine 139. The precise mechanism for the sensitivity of the leukemia and lymphoma cell lines compared to solid cancer cell lines remains to be fully understood. Sensitivity of the hematopoietic cancer cell lines did not correlate with total Chk1 protein expression levels or with the phosphorylation status of Chk1 on serine 296, 317 or 345. This observation is counter to that of Cole *et al. *[20] who identified neuroblastoma as a potential therapeutic target for Chk1 inhibition and that sensitivity to Chk1 inhibition by either siRNA or small molecules correlated with Chk1 S296 phosphorylation. Likewise, our own study in triple-negative breast cancer identified Chk1 S296 and to a lesser extent S317 phosphorylation status as a useful prognostic marker of cell line sensitivity (data not shown).

Previous work by Davies *et al. *[27] identified the selective Chk1 inhibitor, Chk1-A, as anti-proliferative as a single-agent in a range of human cancer cell lines *in vitro*. In this study, they identified several blood-derived cancer cell lines as particularly sensitive to Chk1-A (HEL92.1.7 and Molm13) but overall, the blood-derived cancer lines (average GI_50_ 71 nM, n = 6) were not dramatically more sensitive to Chk1-A than those derived from solid tumors (average GI_50_ 125 nM, n = 7). This is in contrast to that observed with V158411, a novel Chk1 inhibitor structurally distinct from Chk1-A. Hematopoietic-derived cell lines (average GI_50_ 0.17 μM, n = 5) were around 28-fold more sensitive to V158411 compared to cell lines derived from solid cancers (average GI_50_ 4.8 μM, n = 14). As observed in our study, Chk1-A induced a collapse of DNA replication and apoptosis without premature mitosis in the HEL92.1.7 human erythroleukemia cell line [28]. This corresponded with an increase in Chk1 phosphorylation on S345 and pH2AX on S139 and hyper-activation of CDKs. These observations correlate closely with the effect of V158411 single-agent activity in the cell lines utilized in this study. Our work suggests that the mechanism of growth inhibition and cell death observed with Chk1-A in the HEL92.1.7 cell line by Davies *et al.* is applicable to a wider range of blood-derived cancers. The observation that Chk1-A exhibits potent single agent activity in solid cancer cell lines as well as hematopoietic cancer cell lines (in contrast to V158411 and PF-477736) suggests that Chk1-A may inhibit additional kinases important for proliferation and survival of solid cancer-derived cell lines.

The mechanism by which Chk1 inhibition leads to the death of hematopoietic cells is yet to be fully elucidated and understood. The molecular defects in these cell lines most likely occur in pathways for which Chk1 can mutually compensate to protect genomic integrity and therefore Chk1 inhibition is synthetically lethal. Studies in other cancer models provide possible mechanisms which may leave these cell lines more Chk1 dependent than other solid cancer cell types such as lung or colon cancer. Two possible mechanisms have so far been suggested for Chk1 inhibitor sensitivity: increased oncogenic replicative stress or reduced DNA repair capacity due to defects in specific DNA repair pathways especially those responsible for processing and repairing DNA double strand breaks (DSBs) [29,30].

Two previous studies, one in neuroblastoma cells [20] and another in a mouse derived Eμ-myc driven lymphoma cell model [25], identified increased oncogenic replicative stress due to amplification of the *Myc* oncogene as a potential underlying mechanism for sensitivity to Chk1 inhibition. In the Eμ-myc lymphoma model, sensitivity to the Chk1 inhibitor PF-477736 was dependent on a p53 wild type background. Apoptosis induced by oncogenic replicative stress can be suppressed by ATR and Chk1 [29,31]. All the cell lines used in this study, with the exception of MV4-11, are known to harbor amplifications of the c-myc oncogene [32,33] and therefore increased replicative stress due to amplified Myc driven proliferation [34] may underlie the sensitivity of some of these cell lines. However, in contrast to the Eμ-myc lymphoma model, all of the four c-myc amplified sensitive cell lines harbor mutations in p53 suggesting that sensitivity to Chk1 inhibitors may not be dependent on a p53 wild type background. The CML cell line K562 has amplifications in the c-myc and l-myc oncogenes but is resistant, compared to all the other leukemia and lymphoma cell lines so far tested, to Chk1 inhibitors as single agents. Therefore additional factors along with Myc induced oncogenic stress potentially contribute to Chk1 inhibitor sensitivity.

MV4-11 cells harbor an internal tandem duplication (ITD) in the juxtamembrane domain of FLT3 leading to deregulated FLT3 kinase signaling that drives the proliferation of this cell line [35]. Like deregulation of the *c-Myc* oncogene, the FLT3-ITD mutation induces oncogenic replicative stress [36,37] and may account for the sensitivity of this cell line to Chk1 inhibition. Along with U937 and HL-60 cells, MV4-11 cells exhibited a high level of expression of H2AX phosphorylated on serine 139 under normal cell growth conditions. Increased expression of pH2AX (S139) is associated with increased DNA damage especially double strand breaks [38] and in MV4-11 cells is consistent with increased oncogenic replicative stress induced by FLT3 mutation.

Molecular defects in pathways responsible for processing DNA breaks, especially DNA double strand breaks, have been postulated to be potentially synthetically lethal with Chk1 inhibition. One example so far discovered is in the Fanconi Anemia (FA) DNA repair pathway. The Fanconi Anemia (FA) repair pathway is responsible for repairing crosslinked DNA and maintaining chromosomal stability [17]. FA deficient cell lines were found to be sensitive to Chk1 silencing by siRNA and the small molecule Go6975 compared to FA proficient cells due to an accumulation of unrepairable DNA double strand breaks [18]. Similarly, AML with a complex karyotype demonstrate high levels of constitutive DNA damage and checkpoint activation. siRNA against Chk1 or the small molecule kinase inhibitor UCN-01 reduced the clonogenic survival of patient derived AML blast cells [19]. UCN-01 is a non-specific pan-kinase inhibitor derived from staurosporine and effects induced by this molecule cannot be reliably attributed to Chk1 inhibition. Reduced or defective DNA strand break repair capacity could underlie the sensitivity of leukemia and lymphoma cell lines to Chk1 inhibition. The sensitivity of leukemia and lymphoma cell lines to Chk1 inhibition may be due to reduced DNA repair capacity, oncogenic replication stress or a combination of both mechanisms.

All the studies so far conducted have been undertaken on established cell lines that grow indefinitely under optimal culture conditions. Selection of these cell lines for growth in culture may have resulted in the selection for factors that drive cell proliferation in culture rather than tumor proliferation *in situ*. Replicative stress due to deregulated oncogenes, and hence sensitivity to Chk1 inhibitors, may be amplified due to selection of cells that proliferate rapidly in culture and may not truly reflect the oncogenic replicative stress observed in human disease. Further work is needed on leukemia and lymphoma samples derived from patients that have undergone limited *ex vivo* culture to confirm and understand these observations.

From these studies, Chk1 inhibitors may be a useful addition to the arsenal of drugs suitable for use in the clinic against hematopoietic cancers. The ability to stratify patients based on genetic markers predictive of sensitivity will be necessary to achieve optimal clinical benefit. Studies so far suggest that deregulated Myc oncogene expression may be one such marker.

## Conclusions

Cell lines derived from human leukemias and lymphomas exhibited greater sensitive to the Chk1 inhibitor V158411 than cell lines derived from solid tumors. Replication stress, due to oncogene activation, may account for the sensitivity of these cell lines to Chk1 inhibition. This data supports the further evaluation of Chk1 inhibitors in hematopoietic cancers as single agents as well as in combination with standard of care cytotoxic drugs.

## Methods

### Cell culture and cytotoxicity assay

All cells were obtained from the American Type Culture Collection (ATCC) or Leibniz Institute DSMZ-German Collection of Microorganisms and Cell Cultures (DSMZ) and cultured in DMEM or RPMI containing 10% FCS (Invitrogen). The cytotoxicity of V158411 was determined following exposure of cells in 96 well plates to a 10-point titration for 72 hours. Cell proliferation was determined using sulphorhodamine B staining following protein precipitation with 10% TCA for adherent cell lines or cell titer glo (Promega) for suspension cell lines. For cell counts, cells were seeded in 24 well plates and counted daily using a haemocytometer following trypan blue staining. Cells were diluted to maintain log phase cultures.

### Determination of caspase-3/7 dependent apoptosis

Cells were seeded in 96 well plates and treated with 5- or 10-times the GI_50_ of V158411 for 24 or 48 hours. Caspase-3/7 activity was determined using a homogenous caspase-3/7 luminescence kit (Promega).

### Antibodies and western blotting

Anti- pHistone H3 (S10) was obtained from Millipore; Chk1, pChk1 (S317), pChk1 (S345), Cdc2, pCdc2 (Y15), Cyclin B1 and pH2AX (S139) from Cell Signaling Technologies and pChk1 (S296) from Abcam. Treated and untreated cells were washed once with PBS and lysed in 50 mM Tris-pH6.8, 2% SDS, protease and phosphatase inhibitor cocktails (Roche) and boiled for 5 minutes. Protein concentration was determined using BCA kit (Pierce). Equal amounts of lysate were separated by SDS-PAGE and western blot analysis conducted using the antibodies indicated above.

### Flow cytometry

Cells were seeded in 6-well plates and subsequently treated with the indicated concentrations of V158411 for 24 or 48 hours. All cells were harvested, fixed in 70% ethanol and stained with propidium iodide/RNase A. Cell cycle profiles were examined by flow cytometry using a FACSArray cytometer (BD) and FACSDiva software (BD).

## Competing interests

CB, KS and AJM undertook this work as employees of Vernalis R&D Ltd. AJM is a stock option holder of Vernalis R&D Ltd.

## Author’ contributions

AJM designed and coordinated the studies and is the author responsible for writing the paper. CB, KS and AJM carried out the studies. All authors read and approved the final manuscript.

## Author information

All authors are either current or past employees of Vernalis R&D Ltd and undertook this study as part of their employment. AJM is a stock option holder of Vernalis R&D Ltd.
